# Supplementary material for: Structural insights into the conformational changes of BTR1/SLC4A11 in complex with PIP2
Source: Nat Commun. 2023 Oct 3;14:6157. doi: 10.1038/s41467-023-41924-0 (PMC10547724; doi:10.1038/s41467-023-41924-0)
Supplement: Supplementary file 1 — Supplementary Information [file 41467_2023_41924_MOESM1_ESM.pdf]

Supplementary information for

# Structural insights into the conformational changes of BTR1/SLC4A11 in complex with $PIP_2$

Yishuo Lu, Peng Zuo, Hongyi Chen, Hui Shan, Weize Wang, Zonglin Dai, He Xu, Yayu Chen, Ling Liang, Dian Ding, Yan Jin, Yuxin Yin*

*To whom correspondence should be addressed: Yuxin Yin (yinyuxin@hsc.pku.edu.cn)

This file includes:
Supplementary Figs. 1-6
Supplementary Table 1

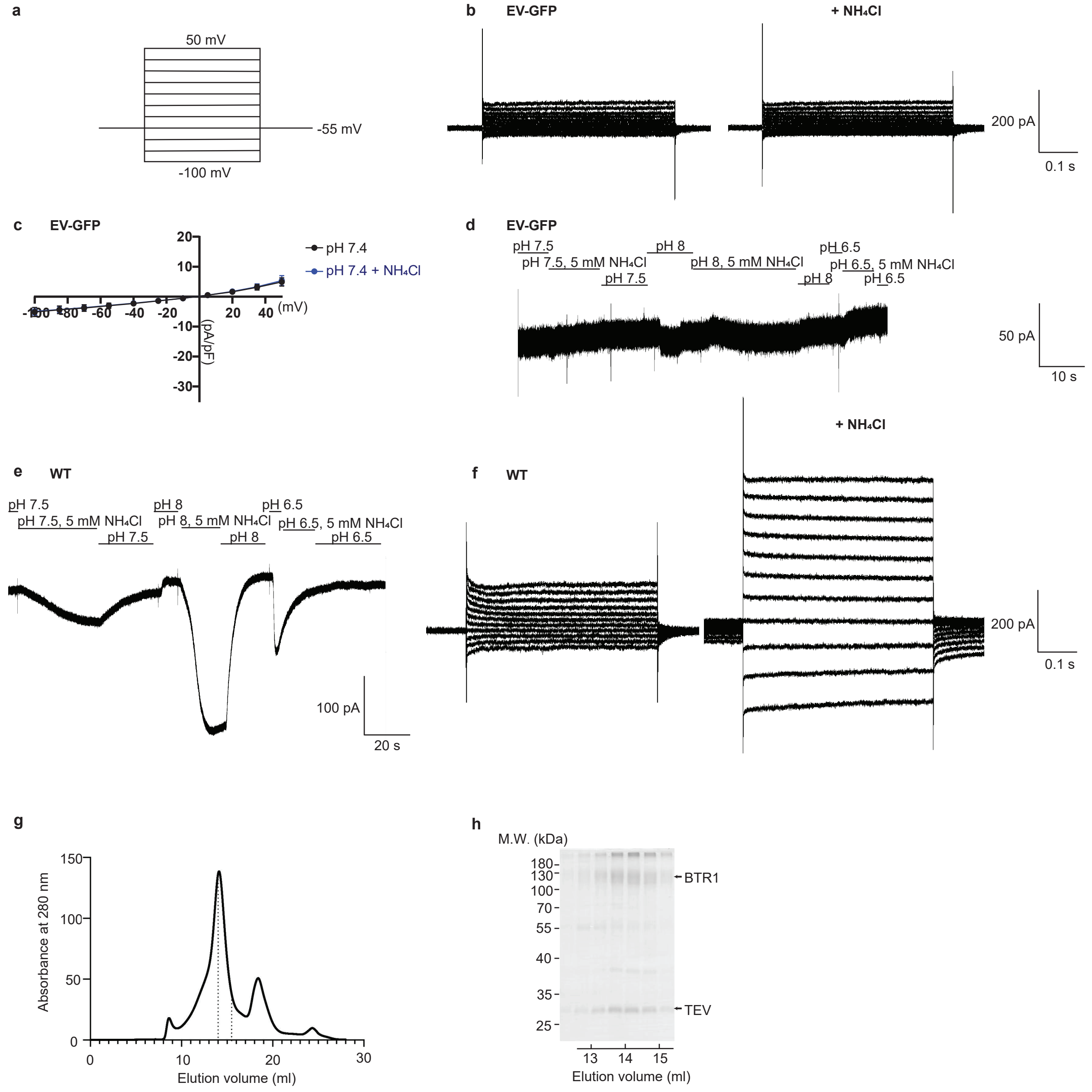


**Supplementary Fig. 1. Characterization of BTR1.**

**a** Schematic diagram of the protocol followed for the I-V curve electrophysiology experiments. The voltage was shifted stepwise from -100 mV to 50 mV with 15 mV step.

**b** Representative whole-cell recording of EV-GFP transfected control cells at pH 7.4 with (right) or without (left) the addition of 5 mM $NH_4Cl$. The scale bar is labeled at the right side.

**c** I-V curve of EV-GFP transfected control cells at pH 7.4 with or without the addition of 5 mM $NH_4Cl$. The current values has been normalized by cell capacitance. Data shown are mean values ± s.d. of n=3 biologically independent experiments.

**d,e** Representative whole-cell recording of EV-GFP transfected control cells (**d**) and wild-type BTR1 transfected cells (**e**) at pH 6.5, 7.4 and 8.0 in the presence or absence of 5 mM $NH_4Cl$.

**f** Representative whole-cell recording of wild-type BTR1 transfected cells at pH 7.4 with (right) or without (left) the addition of 5 mM $NH_4Cl$.

**g** Size exclusion chromatogram of purified BTR1. The fraction indicated by the dotted lines are used for cryo-EM sample preparation.

**h** SDS-PAGE of purified BTR1 after gel filtration.

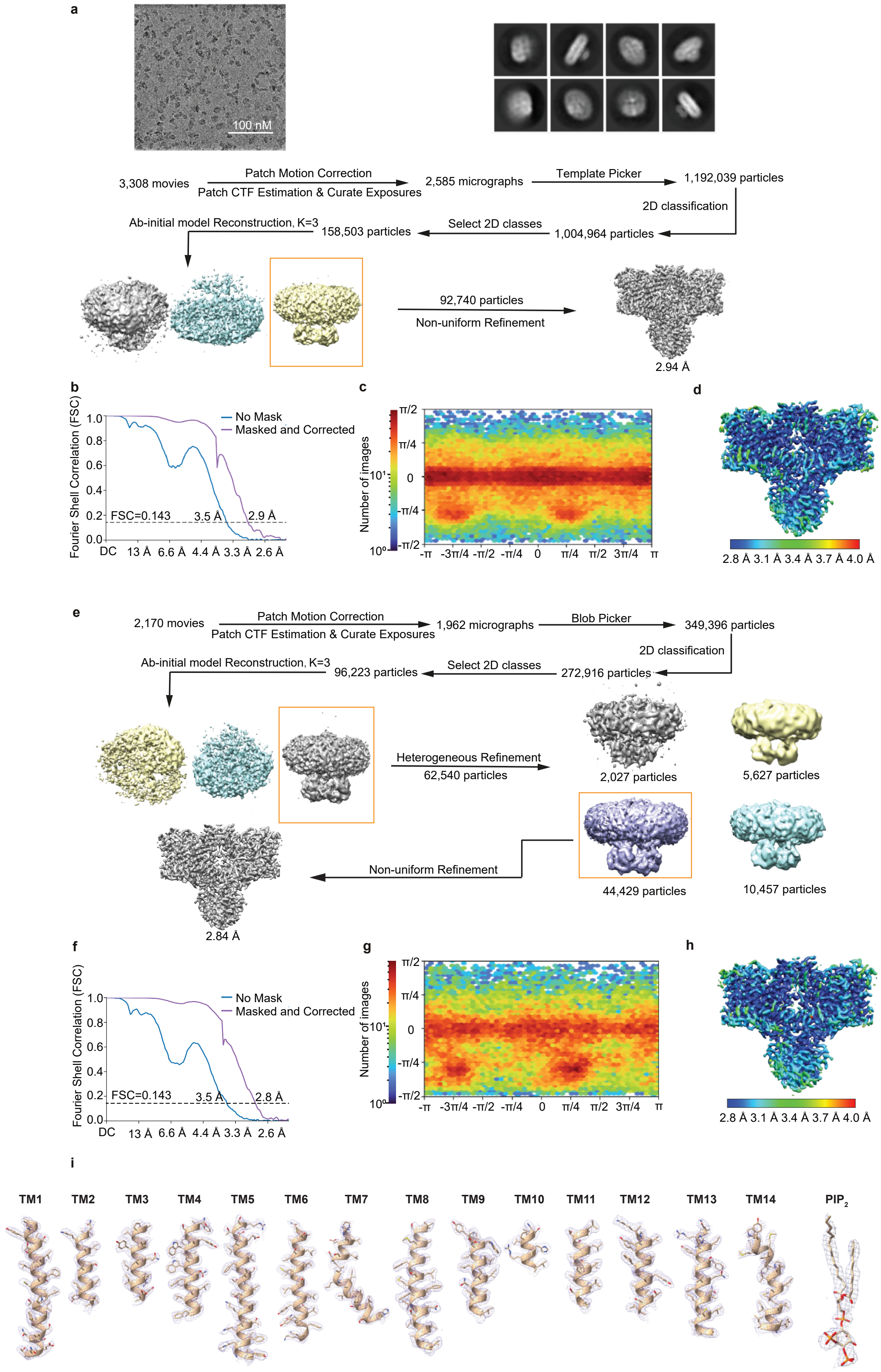


**Supplementary Fig.2. Workflows for cryo-EM processing of BTR1 in the outward-facing conformation ($BTR1_{OF/APO}$ state and $BTR1_{OF/NH_3}$ state).**

**a** Image processing workflow of $BTR1_{OF/APO}$ state structure.
**b** Resolution estimate of $BTR1_{OF/APO}$ state structure based on the criterion of the FSC 0.143 cut-off.
**c** Angular distribution of the final reconstruction of $BTR1_{OF/APO}$ state structure.
**d** Local-resolution estimation of the final density map of $BTR1_{OF/APO}$ state structure within cryoSPARC.
**e** Image processing workflow of $BTR1_{OF/NH_3}$ state structure.
**f** Resolution estimate of $BTR1_{OF/NH_3}$ state structure based on the criterion of the FSC 0.143 cut-off.
**g** Angular distribution of the final reconstruction of $BTR1_{OF/NH_3}$ state structure.
**h** Local-resolution estimation of the final density map of $BTR1_{OF/NH_3}$ state structure within cryoSPARC.
**i** Cryo-EM densities of the transmembrane helices and $PIP_2$ in the $BTR1_{OF/APO}$ state structure which are shown as mesh, superimposed with the corresponding stick models.

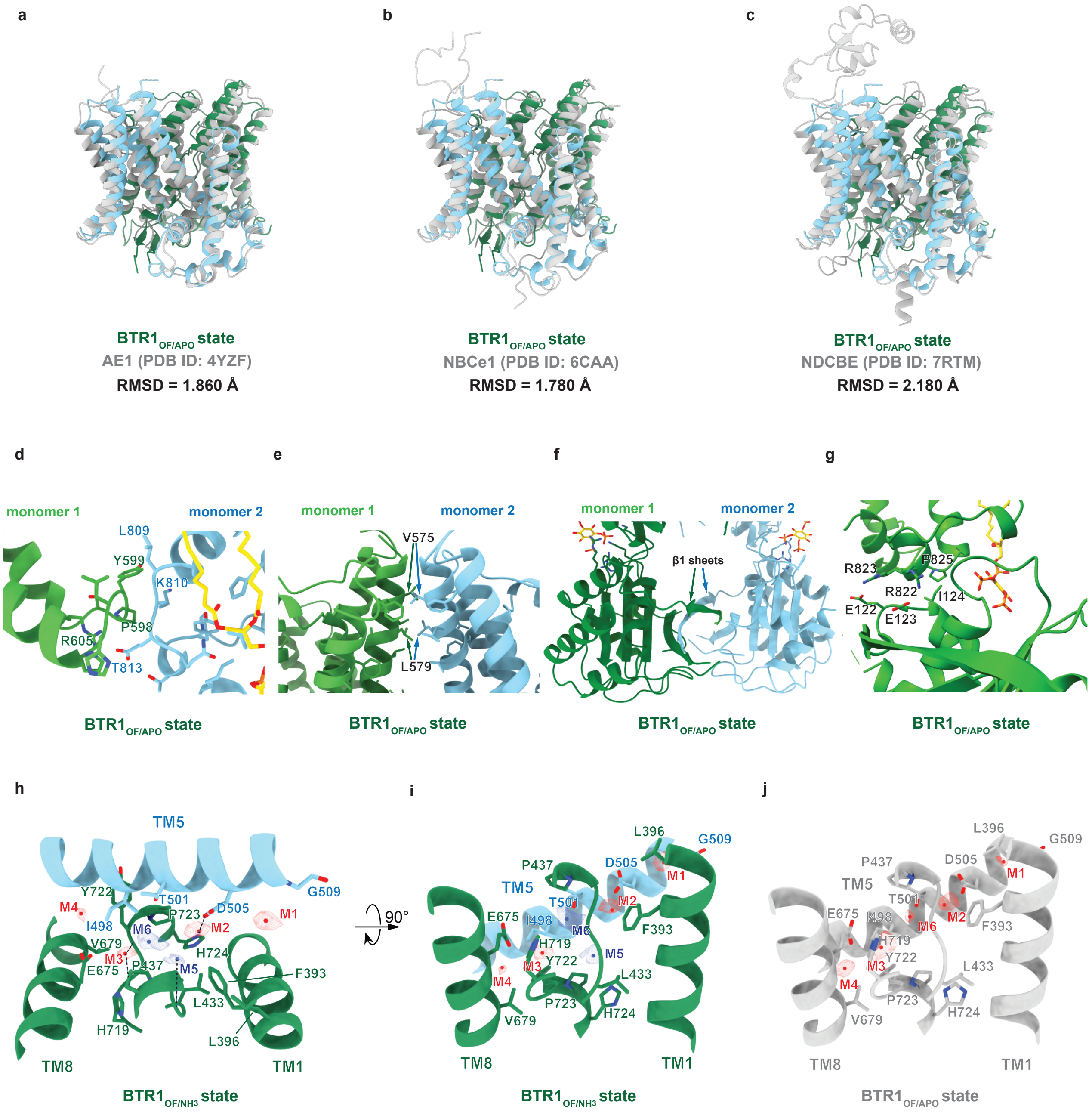


**Supplementary Fig. 3. Close-up views of BTR1 in the outward-facing state.**

**a-c** Structural alignments of the TMDs of BTR1$_{OF/APO}$ with AE1 (PDB ID: 4YZF) (**a**), NBCe1 (PDB ID: 6CAA) (**b**) and NDCBE (PDB ID: 7RTM) (**c**). The RMSDs of the TMD alignments are labeled below.

**d** The interface of the BTR1 TMD dimer near the intracellular region. The two monomers and amino acids above them are colored in green and blue, respectively. The $PIP_2$ molecule is colored in gold.

**e** The interface of the BTR1 TMD dimer near the extracellular region. The two monomers are colored in green and blue. Amino acids involved in the interactions are shown in stick representation.

**f** The interface of the BTR1 NTD dimer between the β sheets.

**g** The interface of the NTD and TMD within a BTR1$_{OF/APO}$ state monomer.

**h,i** Cartoon representation of the ion permeation pathway of BTR1$_{OF/NH3}$ from different views. Transmembrane helices and corresponding amino acids of the core domain and gate domain are colored in green and light blue, respectively. Extra densities are indicated by M1-M6 and densities with relative different intensity between two states are colored in blue. The hydrogen bonds are indicated by dashed lines in (**h**).

**j** The ion permeation pathway of BTR1$_{OF/APO}$ from the same view of (**i**). Extra densities are colored in red.

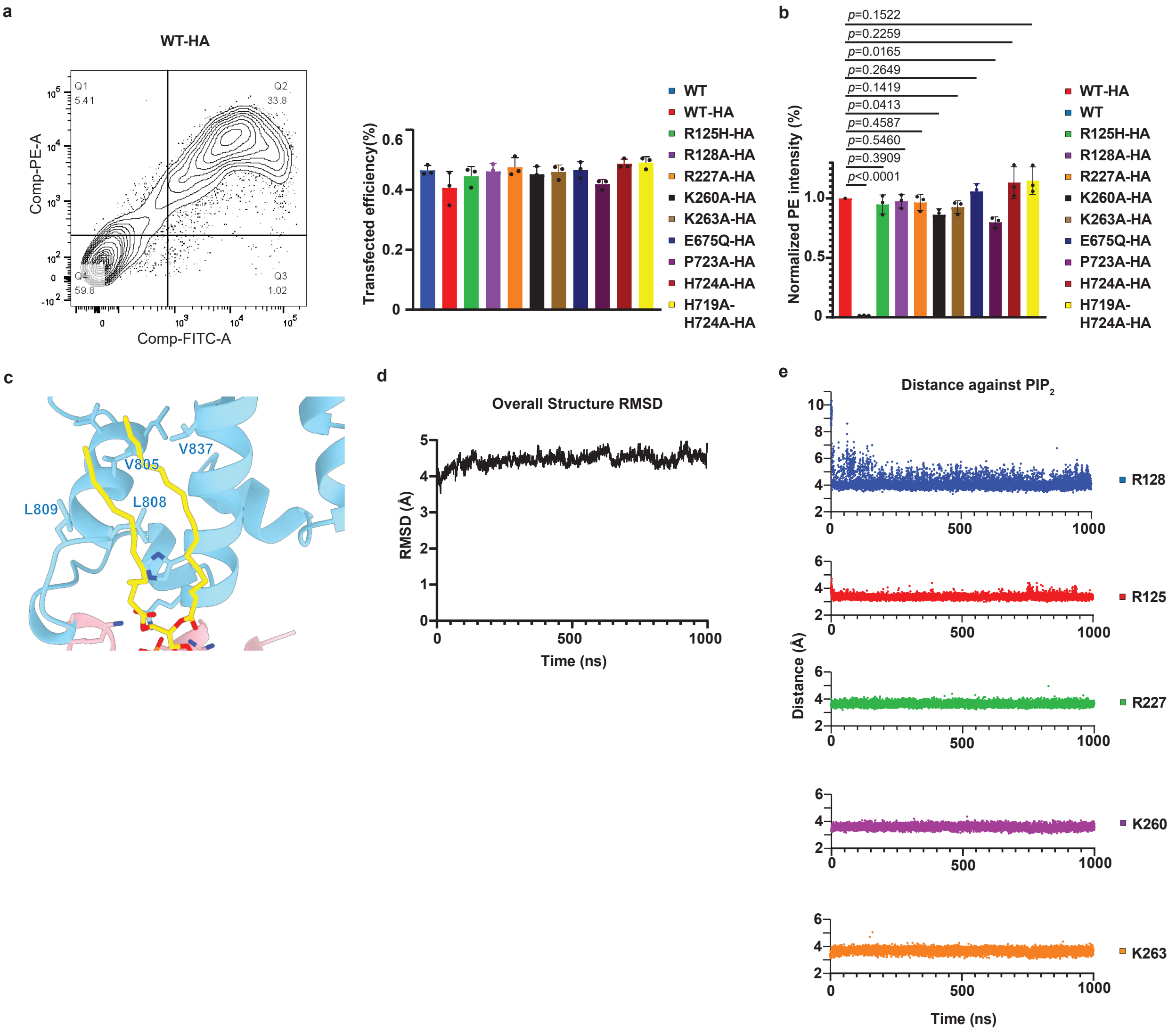


**Supplementary Fig. 4. Membrane expression of BTR1 mutants and $PIP_2$ binding capacity with wild-type BTR1.**

**a** Exemplifying figure of gating strategy and histogram showing the transfection efficiency of various BTR1-HA constructs, which is represented by the percentage of FITC-positive cells in flow cytometry assay. Data shown are means ± s.d. of n=3 biologically independent experiments.

**b** The plasma membrane expression levels of wild-type and mutant HA-tagged BTR1 proteins as determined by flow cytometry analysis. The PE intensities (anti-HA tag) of the mutant BTR1-HA proteins are normalized to that of wild-type BTR1-HA. Data shown are mean values ± s.d. of n=3 biologically independent experiments and *p* values were calculated by two-sided unpaired t-tests.

**c** The hydrophobic interactions between the amino acids of the BTR1 TMD and the phospholipid tails of $PIP_2$. The side chains of the hydrophobic amino acids involved are shown as sticks and $PIP_2$ is colored in gold.

**d** Overall structure RMSD during a 1 µs molecular dynamics simulation. The data points were extracted from AmberTools and VMD.

**e** The relative distance of the nearest hydrogen atoms of 5 basic residues in the $PIP_2$ binding pocket (L site) to the nearest phosphorus atoms of the phosphate groups of $PIP_2$, during a 1 µs molecular dynamics simulation. The data points corresponding to the 5 residues are colored in blue (R128), red (R125), green (R227), purple (K260) and orange (K263), respectively.

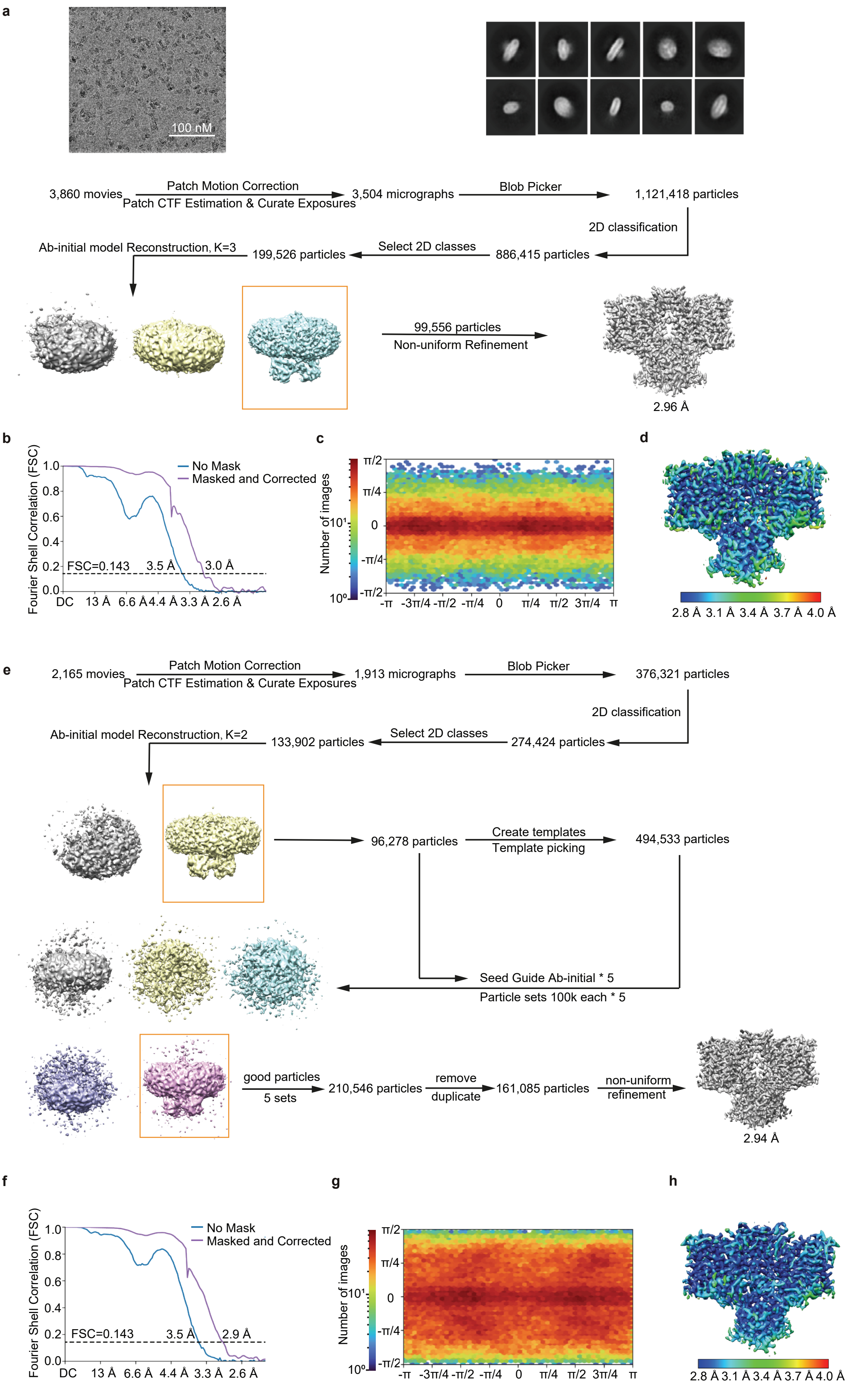


**Supplementary Fig.5. Workflows for cryo-EM processing of BTR1 in the inward-facing conformation ($BTR1_{IF/R125H}$ state and $BTR1_{IF/5.5}$ state).**

**a** Image processing workflows of $BTR1_{IF/R125H}$ state structure.
**b** Resolution estimate of $BTR1_{IF/R125H}$ state structure based on the criterion of the FSC 0.143 cut-off.
**c** Angular distribution of the final reconstruction of $BTR1_{IF/R125H}$ state structure.
**d** Local-resolution estimation of final reconstructed maps determined for $BTR1_{IF/R125H}$ state structure.
**e** Image processing workflows of $BTR1_{IF/5.5}$ state structure.
**f** Resolution estimate of $BTR1_{IF/5.5}$ state structure based on the criterion of the FSC 0.143 cut-off.
**g** Angular distribution of the final reconstruction of $BTR1_{IF/5.5}$ state structure.
**h** Local-resolution estimation of final reconstructed maps determined for $BTR1_{IF/5.5}$ state structure.

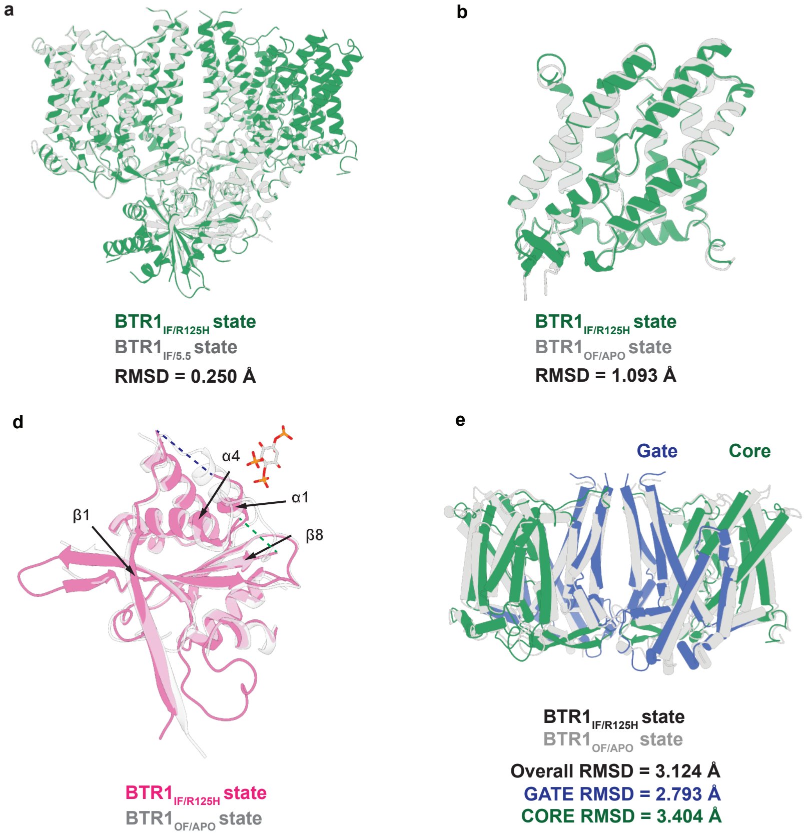


**Supplementary Fig. 6. Structural comparison of BTR1 in the inward-facing state.**
**a** Structural alignment of overall structures of $BTR1_{IF/R125H}$ and $BTR1_{IF/5.5}$. $BTR1_{IF/R125H}$ and $BTR1_{IF/5.5}$ are colored in green and gray, respectively.
**b,c** Structural alignment of the core domains (**b**) and gate domains (**c**) of $BTR1_{IF/R125H}$ and $BTR1_{OF/APO}$. The color schemes are the same as (**a**).
**d** Structural alignment of the NTDs of $BTR1_{IF/R125H}$ and $BTR1_{OF/APO}$. $BTR1_{IF/R125H}$ and $BTR1_{OF/APO}$ are colored in pink and gray, respectively. The flexible loops of $BTR1_{IF/R125H}$ NTD are shown as dotted lines.
**e** Structural comparison of TMDs of $BTR1_{IF/R125H}$ and $BTR1_{OF/APO}$ aligned by the TMDs. The alignment RMSDs are labeled below.

## Supplementary Table 1

## Cryo-EM data collection, refinement and validation statistics

| PDB ID<br>EMDB ID | $BTR1_{OF/APO}$<br>7X1I<br>EMD-32942 | $BTR1_{OF/NH3}$<br>7X1J<br>EMD-32943 | $BTR1_{IF/R125H}$<br>7X1H<br>EMD-32941 | $BTR1_{IF/5.5}$<br>7X1G<br>EMD-32940 |
|---|---|---|---|---|
| **Data collection and processing** | | | | |
| Magnification | 165,000 × | 165,000 × | 165,000 × | 165,000 × |
| Voltage (kV) | 300 | 300 | 300 | 300 |
| Electron exposure ($e^-/Å^2$) | 50 | 50 | 50 | 50 |
| Defocus range (μm) | -1.5 to -1.8 | -1.5 to -1.8 | -1.5 to -1.8 | -1.5 to -1.8 |
| Pixel size (Å) | 0.821 | 0.821 | 0.821 | 0.821 |
| Symmetry imposed | *C2* | *C2* | *C2* | *C2* |
| Initial particle images (no.) | 1,192,039 | 349,396 | 1,121,418 | 376,321 |
| Final particle images (no.) | 92,740 | 44,429 | 99,556 | 161,085 |
| Map resolution (Å) | 2.94 | 2.84 | 2.96 | 2.94 |
| FSC threshold | 0.143 | 0.143 | 0.143 | 0.143 |
| Map resolution range (Å) | 250-2.9 | 250-2.84 | 250-2.96 | 250-2.94 |
| | | | | |
| **Refinement** | | | | |
| Initial model used (PDB code) | AF-Q8NBS3-F1 | AF-Q8NBS3-F1 | AF-Q8NBS3-F1 | AF-Q8NBS3-F1 |
| Model resolution (Å) | 3.0 | 3.0 | 3.1 | 3.1 |
| FSC threshold | 0.5 | 0.5 | 0.5 | 0.5 |
| Model resolution range (Å) | 250-3.0 | 250-3.0 | 250-3.1 | 250-3.1 |
| Map sharpening *B* factor ($Å^2$) | -81.9 | -65.6 | -85.9 | -103.7 |
| Model composition | | | | |
| Non-hydrogen atoms | 10920 | 10928 | 10760 | 10760 |
| Protein residues | 1356 | 1356 | 1352 | 1352 |
| Ligands | 2 | 2 | 0 | 0 |
| *B* factors ($Å^2$) | | | | |
| Protein | 43.69 | 44.33 | 37.65 | 37.65 |
| Ligand | 44.58 | 45.97 | --- | --- |
| R.m.s. deviations | | | | |
| Bond lengths (Å) | 0.004 | 0.004 | 0.004 | 0.004 |
| Bond angles (°) | 0.882 | 0.904 | 0.946 | 0.927 |
| Validation | | | | |
| MolProbity score | 1.25 | 1.20 | 1.18 | 1.19 |
| Clashscore | 4.82 | 4.14 | 3.88 | 4.11 |
| Poor rotamers (%) | 0.34 | 0.85 | 0.17 | 0.17 |
| Ramachandran plot | | | | |
| Favored (%) | 98.80 | 98.04 | 98.19 | 98.11 |
| Allowed (%) | 1.20 | 1.96 | 1.81 | 1.89 |
| Disallowed (%) | 0 | 0 | 0 | 0 |
| Model content | 104-160<br>172-181<br>191-203<br>208-307<br>336-521<br>570-738<br>745-887 | 104-160<br>172-181<br>191-203<br>208-307<br>336-521<br>570-738<br>745-887 | 105-121<br>131-160<br>173-256<br>264-306<br>336-522<br>569-640<br>645-887 | 105-121<br>131-160<br>173-256<br>264-306<br>336-522<br>569-640<br>645-887 |

## Uncropped figures

Supplementary Figure 1h

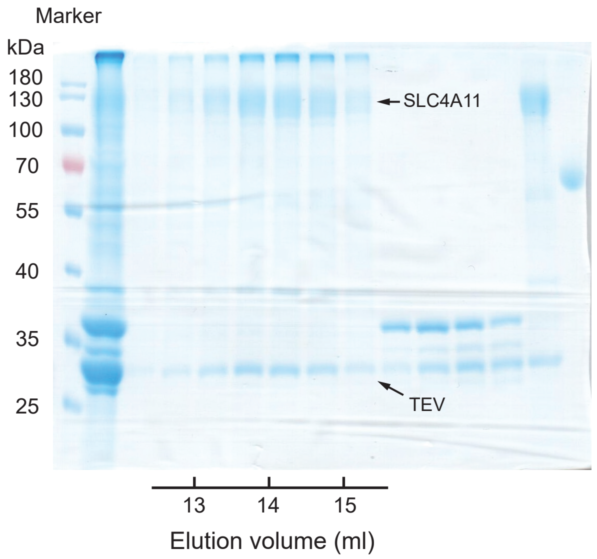

## Primer List

| | |
|---|---|
| hBTR1-F | CAATTACAGCTCTTAAGGAATTCATGAGCCAGGTCGGGGGGCGG |
| hBTR1-R | CTCCCTCGACACCTCCCTCGAGAGGCCTGTGCTCAGCGTCCATG |
| hBTR1-R125H-F | CTTCAAGGAAGAGATCCATGCGCACCGCGACCTAG |
| hBTR1-R125H-R | CTAGGTCGCGGTGCGCATGGATCTCTTCCTTGAAG |
| hBTR1-R128A-F | GAAGAGATCCGTGCGCACGCCGACCTAGATGGCTTCCTG |
| hBTR1-R128A-R | CAGGAAGCCATCTAGGTCGGCGTGCGCACGGATCTCTTC |
| hBTR1-R227A-F | CCATGAAGGCCCTACAGAAGGCGCACGTGTGCATCAGCCGCCTG |
| hBTR1-R227A-R | CAGGCGGCTGATGCACACGTGCGCCTTCTGTAGGGCCTTCATGG |
| hBTR1-K260A-F | GGCCCCACCCAAGATGGCAAGCACTAAGACTGCGATG |
| hBTR1-K260A-R | CATCGCAGTCTTAGTGCTTGCCATCTTGGGTGGGGCC |
| hBTR1-K263A-F | CCCAAGATGAAAAGCACTGCGACTGCGATGGAGGTGGCG |
| hBTR1-K263A-R | CGCCACCTCCATCGCAGTCGCAGTGCTTTTCATCTTGGG |
| hBTR1-E675Q-F | CATGCTCTTCTTCATCCAGCAGAACTTGGTGGC |
| hBTR1-E675Q-R | GCCACCAAGTTCTGCTGGATGAAGAAGAGCATG |
| hBTR1-P723A-F | CTTGGATCCATGCCGCCTACGCCCACTCCCCGCTGCACGTG |
| hBTR1-P723A-R | CACGTGCAGCGGGGAGTGGGCGTAGGCGGCATGGATCCAAG |
| hBTR1-H724A-F | GATCCATGCCGCCTACCCCGCCTCCCCGCTGCACGTGCGAG |
| hBTR1-H724A-R | CTCGCACGTGCAGCGGGGAGGCGGGGTAGGCGGCATGGATC |
| hBTR1-H719A-H724A-F | CTGTTTGGGCTGCCTTGGATCGCTGCCGCCTACCCCGCCTCCCCGC |
| hBTR1-H719A-H724A-R | GCGGGGAGGCGGGGTAGGCGGCAGCGATCCAAGGCAGCCCAAACAG |
| hBTR1-544-HA-545-F | GTCAGGCCTCGGCGCCAGCCTCTACCCATACGATGTTCCAGATTACGCCAACGC CAGCCTCCACACTGCCC |
| hBTR1-544-HA-545-R | GGGCAGTGTGGAGGCTGGCGTTGGCGTAATCTGGAACATCGTATGGGTAGAGG CTGGCGCCGAGGCCTGAC |
