# Supplementary material for: Structural insights into the conformational changes of BTR1/SLC4A11 in complex with PIP2
Source: Nat Commun. 2023 Oct 3;14:6157. doi: 10.1038/s41467-023-41924-0 (PMC10547724; doi:10.1038/s41467-023-41924-0)
Supplement: Supplementary file 3 — Reporting Summary [file 41467_2023_41924_MOESM3_ESM.pdf]

nature portfolio

Corresponding author(s): Yuxin Yin

Last updated by author(s): Sep 11, 2023

Reporting Summary

Nature Portfolio wishes to improve the reproducibility of the work that we publish. This form provides structure for consistency and transparency in reporting. For further information on Nature Portfolio policies, see our Editorial Policies and the Editorial Policy Checklist.

Statistics

For all statistical analyses, confirm that the following items are present in the figure legend, table legend, main text, or Methods section.

n/a	Confirmed	
☐	☒	The exact sample size (*n*) for each experimental group/condition, given as a discrete number and unit of measurement
☐	☒	A statement on whether measurements were taken from distinct samples or whether the same sample was measured repeatedly
☐	☒	The statistical test(s) used AND whether they are one- or two-sided<br>*Only common tests should be described solely by name; describe more complex techniques in the Methods section.*
☒	☐	A description of all covariates tested
☒	☐	A description of any assumptions or corrections, such as tests of normality and adjustment for multiple comparisons
☐	☒	A full description of the statistical parameters including central tendency (e.g. means) or other basic estimates (e.g. regression coefficient) AND variation (e.g. standard deviation) or associated estimates of uncertainty (e.g. confidence intervals)
☒	☐	For null hypothesis testing, the test statistic (e.g. *F*, *t*, *r*) with confidence intervals, effect sizes, degrees of freedom and *P* value noted<br>*Give P values as exact values whenever suitable.*
☒	☐	For Bayesian analysis, information on the choice of priors and Markov chain Monte Carlo settings
☒	☐	For hierarchical and complex designs, identification of the appropriate level for tests and full reporting of outcomes
☒	☐	Estimates of effect sizes (e.g. Cohen's *d*, Pearson's *r*), indicating how they were calculated

Our web collection on statistics for biologists contains articles on many of the points above.

Software and code

Policy information about availability of computer code

Data collection	SerialEM 3.6.11
Data analysis	cryoSPARC, PHENIX, Coot, UCSF Chimera, UCSF ChimeraX, Pymol, GraphPad Prism 9.0.0, Microsoft Excel, HOLE2, FlowJo v10.6.2, pClampfit 10.4, VMD

For manuscripts utilizing custom algorithms or software that are central to the research but not yet described in published literature, software must be made available to editors and reviewers. We strongly encourage code deposition in a community repository (e.g. GitHub). See the Nature Portfolio guidelines for submitting code & software for further information.

Data

Policy information about availability of data

All manuscripts must include a data availability statement. This statement should provide the following information, where applicable:

- Accession codes, unique identifiers, or web links for publicly available datasets
- A description of any restrictions on data availability
- For clinical datasets or third party data, please ensure that the statement adheres to our policy

Cryo-EM maps and atomic coordinates of the BTR1-OF/APO, BTR1-OF/NH3, BTR1-IF/5.5 and BTR1-IF/R125H have been deposited in the EMDB and PDB under the ID codes EMDB: EMD-32942, EMD-32943, EMD-32940, EMD-32941 and PDB: 7X1I, 7X1J, 7X1G, 7X1H, respectively.

Field-specific reporting

Please select the one below that is the best fit for your research. If you are not sure, read the appropriate sections before making your selection.

☒ Life sciences ☐ Behavioural & social sciences ☐ Ecological, evolutionary & environmental sciences

For a reference copy of the document with all sections, see nature.com/documents/nr-reporting-summary-flat.pdf

Life sciences study design

All studies must disclose on these points even when the disclosure is negative.

Sample size	No predetermination of sample size was performed. Sufficient cryo-EM data were collected to achieve adequate map resolutions for model building. The electrophysiological experiments were performed with three or more biological replicates. The sample size was based on previous studies in the field and clearly indicated in the legends.
Data exclusions	Cryo-EM micrographs with ice or ethane contamination, empty carbon, and poor CTF fit (> 5 Å) were excluded manually. Particles belonging to bad classes were discarded and the data processing flowchart were summarized in Extended Data Fig. 2a, 2e, 9a, 9e. These criteria were preestablished and the procedure is a common practise in cryo-EM image analysis. No data was excluded in functional studies.
Replication	All attempts at replication were successful according to the detailed protocol described in the methods section. The numbers of replication were described in figure legends.
Randomization	For cryo-EM 3D refinement, all particles were randomly split into two groups.
Blinding	The investigators were blinded to group allocation during cryo-EM data collection and analysis.

Reporting for specific materials, systems and methods

We require information from authors about some types of materials, experimental systems and methods used in many studies. Here, indicate whether each material, system or method listed is relevant to your study. If you are not sure if a list item applies to your research, read the appropriate section before selecting a response.

Materials & experimental systems

n/a	Involved in the study	
☐	☒	Antibodies
☐	☒	Eukaryotic cell lines
☒	☐	Palaeontology and archaeology
☒	☐	Animals and other organisms
☒	☐	Human research participants
☒	☐	Clinical data
☒	☐	Dual use research of concern

Methods

n/a	Involved in the study	
☒	☐	ChIP-seq
☐	☒	Flow cytometry
☒	☐	MRI-based neuroimaging

Antibodies

Antibodies used	PE anti-HA.11 Epitope Tag Flow Cytometry Antibody (BioLegend, catalog number: 901518)
Validation	Antibodies validation:<br>PE anti-HA.11 Epitope Tag Flow Cytometry Antibody https://www.biolegend.com/en-us/products/pe-anti-ha-11-epitope-tag-antibody-13535

Eukaryotic cell lines

Policy information about cell lines

Cell line source(s)	FreeStyle 293F and Sf9 were from Thermo Fisher Scientific.
Authentication	None of the cell line used was authenticated.
Mycoplasma contamination	All cell lines were tested negative for mycoplasma contamination.
Commonly misidentified lines (See ICLAC register)	No commonly misidentified cell lines were used.

Flow Cytometry

Plots

Confirm that:

☒ The axis labels state the marker and fluorochrome used (e.g. CD4-FITC).

☒ The axis scales are clearly visible. Include numbers along axes only for bottom left plot of group (a 'group' is an analysis of identical markers).

☒ All plots are contour plots with outliers or pseudocolor plots.

☒ A numerical value for number of cells or percentage (with statistics) is provided.

Methodology

Sample preparation	Wild-type BTR1 and its mutants were transfected into HEK293F cells with polyethylenimine (PEI) (Polysciences) at a cell density of 1.5 × 106/ml. Cells were harvested 36 h after transfection. Approximately 2 × 106/ml of each cell type were collected by centrifugation at 1,000 g for 1 min and washed with PBS buffer twice. Cells were then incubated with 50 ul PBS with 1% FBS (PAN Biotech) and 0.1 ul PE anti-HA.11 Epitope Tag Flow Cytometry Antibody (BioLegend) at 37? for 30 min. Cells were washed twice in PBS and resuspended in 500 ul PBS, and then transferred into 5 ml polystyrene round-bottom tubes for flow cytometry analysis.
Instrument	BD LSRFortessa
Software	Flow cytometry data was collected using FACSDiVa software v6.1 (BD Biosciences). Flow cytometry data was analyzed using the FlowJo v10.6.2 software.
Cell population abundance	HEK293F cells were used for assessing the membrane expression of the constructs encoding BTR1 and its mutants. The purity of cells was almost 100%.
Gating strategy	FITC and PE channel were detected, representing the target protein total expression (GFP) and membrane expression (PE-conjugated anti-HA), respectively. The ratio of cell numbers with the two signals were calculated within FlowJo.

☒ Tick this box to confirm that a figure exemplifying the gating strategy is provided in the Supplementary Information.
